# Physical exercise-induced fatigue: the role of serotonergic and dopaminergic systems

**DOI:** 10.1590/1414-431X20176432

**Published:** 2017-10-19

**Authors:** L.M.S. Cordeiro, P.C.R. Rabelo, M.M. Moraes, F. Teixeira-Coelho, C.C. Coimbra, S.P. Wanner, D.D. Soares

**Affiliations:** 1 Laboratório de Fisiologia do Exercício, Escola de Educação Física, Fisioterapia e Terapia Ocupacional, Universidade Federal de Minas Gerais, Belo Horizonte, MG, Brasil; 2 Centro de Formação de Professores, Universidade Federal do Recôncavo da Bahia, Amargosa, BA, Brasil; 3 Laboratório de Endocrinologia e Metabolismo, Instituto de Ciências Biológicas, Universidade Federal de Minas Gerais, Belo Horizonte, MG, Brasil

**Keywords:** Brain, Lethargy, Monoamines, Performance, Physical activity, Reward

## Abstract

Brain serotonin and dopamine are neurotransmitters related to fatigue, a feeling that leads to reduced intensity or interruption of physical exercises, thereby regulating performance. The present review aims to present advances on the understanding of fatigue, which has recently been proposed as a defense mechanism instead of a “physiological failure” in the context of prolonged (aerobic) exercises. We also present recent advances on the association between serotonin, dopamine and fatigue. Experiments with rodents, which allow direct manipulation of brain serotonin and dopamine during exercise, clearly indicate that increased serotoninergic activity reduces performance, while increased dopaminergic activity is associated with increased performance. Nevertheless, experiments with humans, particularly those involving nutritional supplementation or pharmacological manipulations, have yielded conflicting results on the relationship between serotonin, dopamine and fatigue. The only clear and reproducible effect observed in humans is increased performance in hot environments after treatment with inhibitors of dopamine reuptake. Because the serotonergic and dopaminergic systems interact with each other, the serotonin-to-dopamine ratio seems to be more relevant for determining fatigue than analyzing or manipulating only one of the two transmitters. Finally, physical training protocols induce neuroplasticity, thus modulating the action of these neurotransmitters in order to improve physical performance.

## Introduction

Fatigue is a feeling commonly experienced in our daily lives, for example, during periods of vigorous and/or prolonged physical activity. In the sporting context, fatigue is crucial to the performance of athletes in virtually all competitive events, although the determinant factors (either physiological or psychological) for fatigue are specific to the individual events. The present review will focus on fatigue during prolonged (aerobic) exercise, thus characterizing exertion in long-distance sports athletes, including runners, cyclists and swimmers. In these conditions, the determinant factors for fatigue will depend on several aspects, such as exercise intensity and duration, environmental conditions, nutrition and the fitness level of the individual. For instance, regulation of body temperature plays an important role on fatigue during a prolonged exercise (∼60 min) at 60% of the maximal aerobic capacity in hot weather (1).

Fatigue and exhaustion are commonly used as synonymous in the literature, including some studies cited in this review. In general, the moment at which exercise ceases is usually termed as point of exhaustion in human studies. However, the two terms may be related to different processes with distinct physiological characteristics. The feeling of fatigue appears to occur before any damage to body systems, and it is common to see the term ‘volitional fatigue’, indicating that subjects decided to stop exercising. Exhaustion can be defined as extreme fatigue, a state in which an individual may exceed his/her physiological limits and then experience a “catastrophic” failure of homeostasis (2). In this context, the increase of core body temperature would be less in fatigued compared to exhausted individuals. Of note, severe hyperthermia induces some impairments, such as changes in behavior, confusion, loss of coordination and awareness (3), and may favor the occurrence of heat-related disorders (4). In experiments with rats, fatigue is usually defined as the moment when the animals cannot keep the pace on a treadmill during a predetermined time (5). In contrast, exhaustion is confirmed by the observation that exhausted rats lose their righting reflex (i.e., the ability to right themselves when placed on their backs) (5).

The psycho-physiological process that triggers the feeling of fatigue is complex and may result from peripheral and central factors. Peripheral fatigue is defined as the loss of force caused by processes occurring at or distal to the neuromuscular junction (6). In a simpler way, peripheral fatigue can be thought of as fatigue within the muscle itself. Some relevant peripheral factors are specific impairments in neuromuscular transmission and impulse propagation, substrate depletion, reduction in muscle pH, dysfunction within the sarcoplasmic reticulum involving calcium release and uptake, which together impair the ability of muscle fibers to generate power (7).

In the past, fatigue was considered a consequence of the failure of contractile processes in muscle, mainly caused by accumulation of H^+^ ions. However, since the early 2000s, fatigue has been understood as a mechanism that aims to maintain the physiological integrity of the body (8). Signals arising from several systems are integrated in the brain during exercise in order to stop physical exertion or reduce its intensity, as a safety mechanism to prevent the limit of physiological adjustments being exceeded, in any of the systems involved in the exercise (8). Thus, more recent theoretical models for explaining fatigue highlight the involvement of the brain in this process.

The central factors associated with fatigue consist of a number of changes observed in the efferent neurons that alter the recruitment of motor units (9), with some of these changes resulting from altered brain neurochemistry (10). To differentiate central factors from peripheral factors, studies usually compare the individual's ability to generate force voluntarily in relation to the force generated by a supra-maximal electrical stimulus applied to the nerve trunk or intramuscular nerve branches of an active muscle during a voluntary contraction (i.e., the twitch interpolation technique) (11). In these experiments, the evidence for the involvement of central factors on fatigue is provided when force generated by the application of an electrical stimulus exceeds the force generated during voluntary contractions, thereby indicating that some motor units have not been recruited voluntarily. Despite the different concepts involving the process of fatigue (central and peripheral), this classification might be useful only for didactic and methodological issues, because the brain and skeletal muscles have nervous connections between each other that are highly activated during exercise and, therefore, could be relevant for the integration of afferent and efferent signals that modulate fatigue (2).

Several recent studies have investigated the central origin of fatigue, which appears to be associated with the activity of several neurotransmitters, including serotonin (5-HT) (12,13), dopamine (DA) (14,15), acetylcholine (16), angiotensin II (17), noradrenaline (NA) (18,19) and nitric oxide (20). However, considering the emphasis given to the involvement of 5-HT and DA in the development of fatigue in studies with humans or laboratory rodents, this review will focus on the role of these two neurotransmitters.

## Serotonin

Serotonin (5-hydroxytryptamine; 5-HT) is a neurotransmitter synthesized from the amino acid tryptophan (TRP), which is transported through the blood-brain barrier by a specific carrier and is then hydroxylated by the action of tryptophan hydroxylase; this hydroxylation is the rate limiting step in the biosynthesis of 5-HT. Increased plasma levels of free TRP favor increases TRP concentrations in the central nervous system (CNS), and thus any condition that increases this amino acid in the plasma will induce increased concentrations in the CNS and hence the central biosynthesis of 5-HT (21).

The bodies of serotonergic neurons are located in the CNS structures called the raphe nuclei. These nuclei are divided into caudal raphe, with descending projections to the spinal column, and rostral and medial raphe, which send ascending projections to various brain regions, such as the substantia nigra pars compacta (SNpc), thalamus, striatum, nucleus accumbens, hippocampus and hypothalamus. The release of 5-HT into the synaptic cleft leads to the binding of the neurotransmitter to one of its fifteen receptors (divided into 7 families), thus triggering physiological responses (22).

Experiments conducted in mice have provided evidence that exercise changes the TRP levels, as brain concentrations of TRP were increased after swimming to fatigue (23). The first direct evidence of the involvement of 5-HT in modulating fatigue was provided by studies that observed increases in TRP concentrations in both the plasma and brain, accompanied by increased 5-HT concentrations in the brain of rats subjected to moderate intensity exercise (24,25). The “central fatigue hypothesis” was proposed with 5-HT as the modulator of fatigue (26), because increased CNS concentrations of this neurotransmitter during exercise would promote increased lethargy and higher perceived exertion, likely by modifying the tolerance to pain or discomfort, which would limit mental and physical performances (9). Since then, to better understand the “central fatigue hypothesis”, different nutritional and pharmacological manipulations have been carried out in different experimental models to increase or decrease 5-HT concentrations in the CNS. In humans, the nutritional and pharmacological treatments are given peripherally, usually by oral ingestion of supplements or drugs, which can be a confounding factor that would explain the different results obtained in different studies, as the gastrointestinal tract expresses 5-HT receptors (22) and may be the first site affected by these treatments.

Pharmacological manipulations of the activity of 5-HT in the CNS induced changes in physical performance, which supports the theory of participation of this neurotransmitter in the central fatigue mechanisms. In studies with exercising rats, administration of drugs that increase serotonergic activity (agonists of 5-HT receptors) decreased performance, while inhibitors of serotonergic activity (receptor antagonists) increased performance (Table 1). Such changes in performance were not accompanied by peripheral changes in a range of variables including muscle and liver concentrations of glycogen and blood concentrations of glucose (27,28). These findings suggest that the changes in performance are probably due to the action of drugs on the 5-HT system in the CNS.

**Table 1.** Impact of different pharmacological/nutritional manipulations of the serotonergic system on physical performance in laboratory rodents.

Study	Manipulation	Exercise protocol	Performance
Bailey et al., 1993 27	*ip* injection of 1.0 mg/kg of quipazine dimaleate (a 5-HT agonist) or 1.5 mg/kg of LY 53857 (a 5-HT antagonist), immediately before the exercise	Exhausting, constant-speed treadmill running at 20 m/min (5% grade)	quipazine dimaleate: ↓LY 5385: ↑
Calders et al., 1997 50	*ip* injection of 30 mg of BCAA 5 min before the exercise. BCAA prevent the entry of free L-TRP into the brain and, thus, decrease the brain synthesis of 5-HT	Exhausting, constant-speed treadmill running at 20 m/min (8% grade)	↑
Min et al., 2003 33	*ip* injection of Red ginseng (Paeonia radix) extract, which reduces the number of 5-HT-positive cells in the dorsal raphe, once a day for 5 consecutive days. The ginseng was given at three different doses: 10, 50, and 100 mg/kg	Exhausting, constant-speed treadmill running at 20 m/min that was performed on the 5th day of the experiment	10 mg/kg: ↑50 mg/kg: ↑100 mg/kg: ↑ (a dose-dependent effect)
Soares et al., 2003 13; 2004 30; 2007 29	*icv* injection of 20.3 µM of L-TRP, a precursor for 5-HT synthesis, immediately before the exercise	Fatiguing, constant-speed treadmill running at 18 m/min (5% grade) at 23 ± 2°C	↓
Rodrigues et al., 2009 16	*icv* injection of 5×10-3 M of physostigmine, a drug that blocks the running-induced increase in 5-HT in the preoptic area, immediately before the exercise	Fatiguing, constant-speed treadmill running at 20 m/min (5% grade) at 23 ± 2°C	↔
Leite et al., 2010 32	*icv* injection of 60 nmol of losartan immediately before the exercise. This drug increases the 5-HT-to-DA ratio in the hypothalamus.	Fatiguing, constant-speed treadmill running at 18 m/min (5% grade) at 22 ± 2°C	↓
Falavigna et al., 2012 51	Trained rats ingested a diet supplemented with 3.57% BCAA or 4.76% BCAA during ∼6 weeks	Swimming exhaustion test, with a water temperature of 32°C	3.57%: ↑4.76%: ↓
Cordeiro et al., 2014 12	*ip* injections of 100 mg·kg-1·day-1 of p-CPA, a drug that selectively depletes cerebral 5-HT, on each of the three days before the trial. These *ip* injections were associated with an *icv* injection of saline or 20.3 µM of L-TRP immediately before the exercise	Fatiguing, constant-speed treadmill running at 18 m/min (5% grade) at 23°C	ip p-CPA + icv saline: ↔ip p-CPA + icv TRP: ↔

5-HT: serotonin; BCAA: branched-chain amino acids; DA: dopamine; *icv*: intracerebroventricular; *ip:* intraperitoneal; L-TRP: L-tryptophan; p-CPA: *para*-chlorophenylalanine; ↔: no changes in physical performance; ↑: improved performance; ↓: impaired performance.

**Figure 1.**
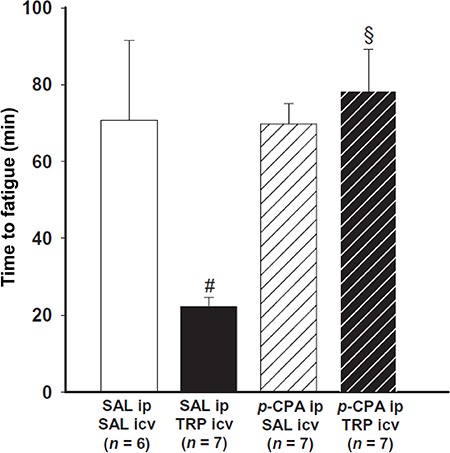
The pharmacological blockade of serotonin synthesis prevents the reduction in physical performance induced by central administration of the serotonin precursor, tryptophan (TRP). The figure shows the effect of intracerebroventricular (*icv*) injections of TRP or saline (SAL) on time to fatigue in rats pretreated with intraperitoneal (*ip*) SAL or p-chlorophenylalanine (*p*-CPA) and that underwent submaximal physical exercise until they were fatigued. Data are reported as means±SE. The number of animals is indicated in parentheses. ^#^P<0.05, significantly different from SAL *ip* + SAL *icv*; ^§^P<0.001, significantly different from SAL *ip* + TRP *icv*. This figure is reprinted with permission from Cordeiro et al., 2014 (12).

The involvement of 5-HT in the fatigue process has been studied by our research group since the early 2000s and our experiments confirm the involvement of this neurotransmitter in the modulation of fatigue. Increasing the levels of central TRP by amino acid injection directly into the cerebral ventricles reduces the time to fatigue in rats subjected to moderate intensity treadmill running (12,16,29,30). The performance reduction caused by intracerebroventricular (icv) TRP was remarkable, and the exercise duration was 60–70% lower after TRP administration, compared with the controls (Figure 1) (12,30).

To confirm that TRP administration reduced physical performance by stimulating 5-HT synthesis, we blocked the action of tryptophan hydroxylase with systemic administration of the inhibitor *para*-chlorophenylalanine (*p*-CPA). Rats treated with *p*-CPA showed no reduction of the time to fatigue after icv administration of TRP, confirming that the ergolytic action caused by increased central TRP was a consequence of the conversion of the amino acid to 5-HT, and excluding the direct participation of this amino acid in the modulation of fatigue (Figure 1) (12).

**Figure 2.**
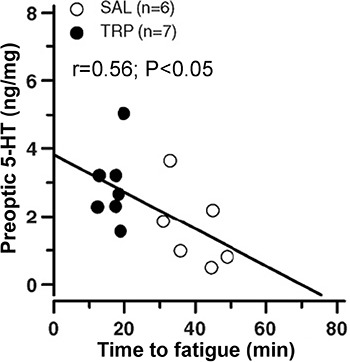
Physical performance is associated with central concentrations of serotonin (5-HT) in rats. The figure shows the significant correlation between the time to fatigue and 5-HT concentrations in the preoptic area in rats that received an intracerebroventricular injection of 2 µL tryptophan (TRP, black circles) or saline (SAL, white circles). This figure is reprinted with permission from Soares et al., 2007 (29).

In addition to its direct effects on behavior, 5-HT can modulate fatigue by changes in regulation of body temperature. Increased central TRP availability reduces mechanical efficiency in rats, leading to an increase in the heat storage rate (i.e., the speed at which heat is stored in the body core) and a reduction in time to fatigue (13,30). Indeed, increased 5-HT concentration in the preoptic area, a brain area that controls the autonomic thermoeffectors (31), induces higher heat storage rate and reduces performance following icv injection of TRP (Figure 2) (29). These findings were corroborated by blocking the angiotensin AT_1_ receptors in the CNS, which raised the concentration of 5-HT in the preoptic area, increased the heat storage rate and lowered the performance of rats (32). On the other hand, stimulation of the central cholinergic system increased cutaneous heat dissipation in rats, attenuating hyperthermia induced by exercise and this lower thermal strain was related to a decrease in 5-HT concentration in the preoptic area (16). Together, these results suggest that 5-HT, acting in the preoptic area, can accelerate exercise cessation, by modulating thermoregulatory mechanisms.

Interestingly, when evaluating the effects of treatment with p-CPA (12), we noticed that rats began to dissipate heat through the skin more rapidly and exhibited less increase of core temperature during exercise. Surprisingly, the lowest thermoregulatory strain caused by the decreased central synthesis of 5-HT was not associated with an increased performance (12). These data are not in agreement with the results obtained in studies that subjected rats to acupuncture or treated them with medicinal plants. The use of such treatments induced ergogenic effects that were associated with decreased brain 5-HT metabolism (33,34).

All the findings reported so far have been obtained with laboratory rodents, which represent a powerful experimental model to manipulate brain neurochemistry by locally administering drugs with agonist and antagonist effects. However, methodological and ethical issues limit the understanding of the involvement of central neurotransmitters in fatigue in humans. Even modern experimental approaches, such as positron emission tomography and near-infrared spectroscopy, fail in revealing the phenotype of the activated neurons, despite the fact that these approaches allow the evaluation of active brain areas in humans under several conditions. Due to ethical issues, it is not allowed to administer drugs and amino acids directly into the brain of humans; therefore, such substances have to be given systemically, which adds a number of confounding factors in studies, including intestinal absorption of administered substances, the degradation of these substances in the vascular periphery and the ability of these substances to cross the blood-brain barrier. As a result of the many confounding factors, studies using dietary manipulations to increase the availability of central TRP in humans show conflicting results regarding physical performance, with reports of increases (35) or no change (36) in performance (Table 2). Nevertheless, human studies are still essential to determine whether the findings obtained in studies in mice or rats are indeed applicable to human physiology.

**Table 2.** Impact of different pharmacological/nutritional manipulations of the serotonergic system on physical performance in humans.

Study	Manipulation	Exercise protocol	Performance
Segura and Ventura, 1988 35	Ingestion of 2 capsules, each containing 150 mg of L-TRP, on the night before the test, at breakfast, lunch time and 1 h before the test	Exhausting, constant-speed treadmill running at 80% VO2MAX; ambient temperature was set at 26°C	↑
van Hall et al., 1995 36	Ingestion of drinks that contained L-TRP (3 g/L) or BCAA at two doses (6 and 18 g/L). These drinks were ingested during exercise	Cycling at a constant intensity that corresponded to 70-75% of the WMAX until exhaustion	L-TRP–3 g/L: ↔BCAA–6 g/L: ↔–18 g/L: ↔
Struder et al., 1998 42	Ingestion of 21 g and 7 g of BCAA 15 min before the test and after 60 min of exercise, respectively, or ingestion of 20 mg of paroxetine, a selective 5-HT reuptake inhibitor, 5 h before the test	Cycling at constant intensity (256.0 ± 19.5 W) that corresponded to a blood lactate level of 2.0 mmol/L until fatigue	BCAA: ↔Paroxetine: ↓
Meeusen et al., 2001 44	Ingestion of 2 capsules containing 20 mg of fluoxetine, a selective 5-HT reuptake inhibitor, on the night before and the morning of the test	A time trial that required the subjects to cycle a predetermined amountof work (equal to 90 min at 65% WMAX)	↔
Roelands et al., 2009 45	Ingestion of 2 capsules containing 10 mg of citalopram, a selective 5-HT reuptake inhibitor, on the evening before and the morning of the test	A time trial that required the subjects to cycle a predetermined amount of work equal to 30 min at 75% WMAX; this exercise was performed at 18°C (temperate) and 30°C (hot conditions)	18°C: ↔30°C: ↔
Teixeira-Coelho et al., 2014 43	Subjects with lower and higher aerobic capacities ingested a capsule containing 10, 20, or 40 mg of paroxetine 4.5 h before the test	Cycling at a constant intensity that corresponded to 70-75% of the maximal power output. Ambient temperature was controlled at 21.4°C	Lower: ↔ (3 doses)Higher:–10 mg: ↔–20 mg: ↓–40 mg: ↔

5-HT: serotonin; BCAA: branched-chain amino acids; L-TRP: L-tryptophan; VO_2MAX_: maximal oxygen uptake; W_MAX_: maximal workload; ↔: no changes in physical performance; ↑: improved performance; ↓: impaired performance.

As it is not possible to directly measure the activity of neurotransmitters in humans, the blood concentrations of some pituitary hormones [prolactin (PRL), adrenocorticotropin, and growth hormone] have been used as indicators of CNS neurotransmitter activity, as both the 5-HT and DA modulate the secretion of these hormones (37). Elevations in plasma concentrations of PRL were observed in individuals performing prolonged exercise in a hot environment, but not in a temperate environment (38). This increase in plasma concentration of PRL suggests increased serotonergic activity and/or reduction of the dopaminergic activity in response to increased core temperature in the hot environment (39). Such changes are likely to occur in the hypothalamus, as 5-HT stimulates and DA inhibits the release of PRL from the anterior pituitary lactotrophs (40). Moreover, increases in serum concentrations of 5-HT and PRL were observed after incremental-intensity exercise to fatigue, both in temperate and hot/humid environments. There was an inverse correlation between the increase in 5-HT and physical performance in the warm environment, indicating a possible role of this neurotransmitter in the fatigue process, primarily in conditions of environmental heat stress (41). However, pharmacological manipulations to change the brain 5-HT concentrations in humans have produced divergent responses, with some studies showing a decrease in performance after administration of selective serotonin reuptake inhibitors (42,43), while other studies have not observed this effect (44,45).

Not only higher 5-HT concentrations, but also the sensitivity of receptors stimulated by 5-HT can modulate feelings of fatigue during prolonged exercise. It has been hypothesized that aerobic training modulates sensitivity of 5-HT receptors in laboratory rodents (46) and humans (47,48), causing desensitization (downregulation) of these receptors. Notably, this decreased sensitivity in rats was more evident in response to vigorous physical training compared to moderate training (46). The findings of two of these three studies (46,47) suggest that trained people may be more resistant to fatigue not only by genotypic characteristics, but also by decreased receptor sensitivity to 5-HT, among other factors. This reduction in sensitivity to 5-HT and the mechanisms involved in the process are still not fully elucidated, mainly due to methodological difficulties related to the study of monoamines in the CNS in humans. Moreover, it is important to note that most studies have investigated sensitivity changes of serotonergic activity of trained individuals when they were at rest, indicating the need to evaluate this response while exercising.

**Figure 3.**
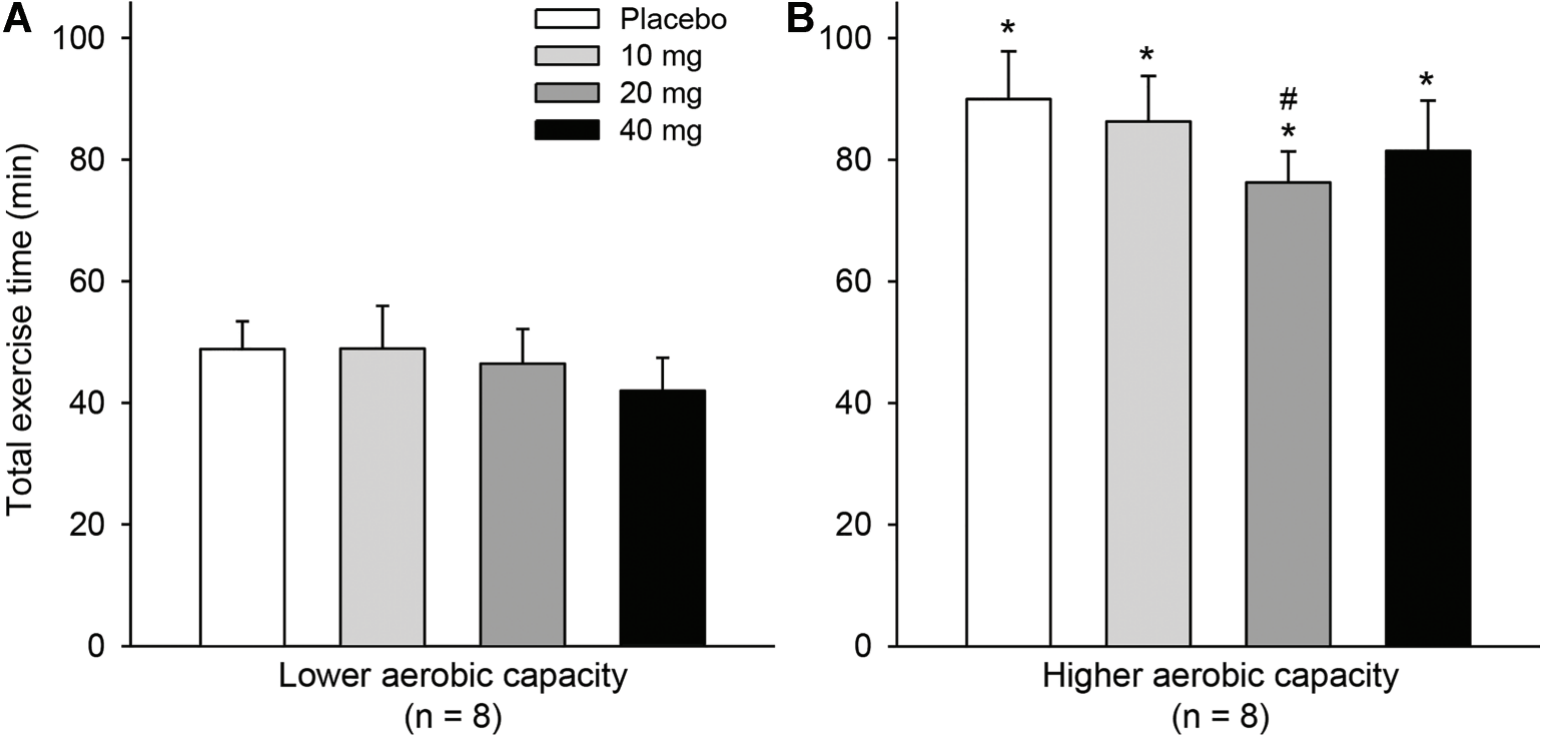
The inhibition of serotonin reuptake affects physical performance in the subjects with higher aerobic capacity but not in those with lower aerobic capacity. The figure shows the time to fatigue by the subjects with lower (*panel A*) and higher (*panel B*) aerobic capacity during cycling at 60% of their maximal power output. Each subject participated in four experimental trials with the following drug conditions: placebo and 10, 20, and 40 mg of paroxetine. Data are reported as means±SE. *P<0.05, significantly different from individuals with low aerobic capacity. ^#^P<0.05, significantly different from the placebo. This figure is reprinted with permission from Teixeira-Coelho et al. 2014 (43).

A recent study in our laboratory investigated the influence of aerobic capacity on the relationship between the central serotonergic activity and fatigue during prolonged exercise in humans. Contradicting the results reported for subjects at rest, pharmacological stimulation of the serotonergic system decreased the time to fatigue in volunteers with higher aerobic capacity compared to the placebo condition, while stimulation of the serotonergic system did not affect the time to fatigue in the group with lower aerobic capacity (Figure 3) (43). The results of this study suggest that the serotonergic activity of individuals with higher aerobic capacity does not have an attenuated response during exercise compared to the activity of individuals with lower aerobic capacity.

Relying on the “central fatigue hypothesis”, several studies have tried to delay fatigue by preventing the increase of 5-HT in the CNS. One of the main strategies used for this purpose is nutritional supplementation with branched-chain amino acids (BCAA). Supplementation of these amino acids reduces entry of free TRP into the CNS, because the BCAA and TRP compete for the same transport system across the blood-brain barrier. In a recent study, supplementation with BCAA before exercise performed to fatigue tended to reduce the concentration of 5-HT in blood samples in the treated group compared to the control group (49). In rats, intraperitoneal treatment with BCAA prior to exercise increased the time to fatigue (50). Interestingly, Falavigna et al. (51) observed that the effect of BCAA on time to fatigue appears to be dose-dependent, as ingestion of smaller and larger quantities of the supplement improved and reduced performance, respectively (Table 1). The higher BCAA dose promoted hyperammonemia, which explains the reduction in performance. Regarding data obtained with humans, some studies have shown that BCAA intake can influence the physical and mental performances of healthy individuals (51–55). In contrast, athletes who were supplemented with amino acids, including BCAA, before and during participation in an ultramarathon (100 km) showed no improvement in performance (56). This result is corroborated by several other studies that have shown no effects on fatigue induced by supplementation with these amino acids during an incremental-intensity exercise in a temperate environment (57) or prolonged exercise in temperate (36,42) and warm (58,59) environments.

In summary, studies in rats and humans provide evidence that central serotonergic activity is related to fatigue during prolonged exercise. However, the results obtained in human studies are still quite controversial and many issues need clarification, such as the mechanisms underlying the physiological responses modulated by 5-HT and the effects of physical training on the activity/sensitivity of the serotonergic system.

## Dopamine

Dopamine (DA; 3,4-dihydroxy-phenylethylamine) is another neurotransmitter involved in the central fatigue mechanisms. The first evidence of the association between DA and exercise dates from the 1970s and 1980s in studies with rats. Peripheral administration of amphetamine, a DA releaser, increased the time to fatigue (60), whereas neuronal injury in dopaminergic pathways reduced performance (61).

DA is synthesized from the amino acid tyrosine that crosses the blood-brain barrier, is transformed into L-3,4-dihydroxyphenylalanine (L-DOPA) by tyrosine hydroxylase and then, to DA by dopadecarboxylase. The conversion mediated by tyrosine hydroxylase, which is stimulated by calcium, is considered the limiting step of the synthesis of this monoamine (62). The main dopaminergic efferent neurons originate in the SNpc and ventral tegmental area (VTA) with projections to striatal structures, and to cortical, limbic and hypothalamic areas. The major efferent pathways are the nigrostriatal (SNpc projections to the striatum), the mesocorticolimbic (VTA projections to the cortical and limbic regions) and the nigro-hypothalamic (SNpc projections to the hypothalamus) pathways (63). DA is released from the terminal nerve and binds to one of five DA receptors, which are divided into two families: D_1_-like (containing D_1_ and D_5_ receptors) and D_2_-like (with D_2_, D_3_ and D_4_ receptors) (62).

The augmented activity of the dopaminergic system in response to exercise initiation appears to be due to an increase in the central levels of calcium, which increases the activity of tyrosine hydroxylase through the activation of the calcium-calmodulin system (64). In contrast, the decrease in DA concentration that occurs as exercise continues likely results from the inhibitory effects of 5-HT. Evidence indicates that the activity of dopaminergic system is related to the development of fatigue through modulation circuits associated with the thermoregulatory and motor control as well as motivation and reward mechanisms (37,65,66).

During exercise, the increase in the activity of the dopaminergic system in the preoptic area seems to influence tolerance to heat stress. In rats, both the icv administration of DA (15), and the intraperitoneal injection of bupropion – a dual DA/NA reuptake inhibitor (67) – resulted in ergogenic effects (Table 3). In these experiments, the intensification of the dopaminergic activity allowed the rats to tolerate higher core temperatures before stopping exercise. It is noteworthy that the reverse is also true as rats treated with DA antagonists seemed to tolerate a smaller increase in core temperature and therefore exhibited a lower time to fatigue (68).

**Table 3.** Impact of different pharmacological/nutritional manipulations of the dopaminergic system on physical performance in both laboratory rodents and humans.

Study	Manipulation	Exercise protocol	Performance
**Laboratory rodents**			
Gerald, 1978 60	*ip* injection of 2 doses of amphetamine, a DA releaser, prior to exercise	Exhausting, constant-speed (10.7-26.8 m/min, 8% grade) treadmill running	2.5 mg/kg: ↑10.0 mg/kg: ↓
Heyes et al., 1985 61	*ip* injection of different doses of apomorphine, a non-selective DA agonist	Exhausting, constant-speed (36.0 m/min, 0% grade) treadmill running	1 mg/kg: ↔2 mg/kg: ↑
Hasegawa et al., 2008 67	*ip* injection of 17 mg/kg of bupropion, a dual DA/NA reuptake inhibitor, 20 min before the exercise	Exhausting, constant-speed (26 m/min) treadmill running at 30°C	↑
Balthazar et al., 2009 15	*icv* injection of 5×10-3 M (10 nmol) of DA solution immediately before the exercise	Fatiguing, incremental-speed running: initial speed of 10 m/min (5% grade), which was increased by 1 m/min every 3 min at 22 ± 1°C	↑
Balthazar et al., 2010 68	*icv* injection of 5×10-3 M (10 nmol) of SCH-23390, a D1 antagonist or 5×10-3 M (10 nmol) of Eti, a D2 antagonist immediately before the exercise	Fatiguing, incremental-speed running: initial speed of 10 m/min (5% grade), which was increased by 1 m/min every 3 min at 22 ± 2°C	SCH-2239: ↓Eti: ↓
Zheng et al., (2016) 69	*ip* injection of 10 mg/kg caffeine 60 min before the exercise. Caffeine promotes DA release in the preoptic area and anterior hypothalamus	Fatiguing, constant-speed (18 m/min, 5% grade) treadmill running at 23°C	↑
**Humans**			
Watson et al., 2005 14	Ingestion of 2 capsules containing 300 mg of bupropion: one on the night before and the other taken upon waking on the morning of the trial	A time trial that required the subjects to cycle a predetermined amount of work equal to 30 min at 75% WMAX; this exercise was performed at 18°C (temperate) and 30°C (hot conditions)	18°C: ↔30°C: ↑
Roelands et al., 2008 70	Ingestion of a capsule containing 20 mg of methylphenidate, a DA reuptake inhibitor, 1 h before the start of trial	Same exercise protocol as in Watson et al. (2005) 14. The exercise was performed at 18°C and 30°C	18°C: ↔30°C: ↑

5-HT: sertononin; DA: dopamine; Eti: eticlopride solution; *icv*: intracerebroventricular; *ip*: intraperitoneal; NA: noradrenaline; W_MAX_: maximal workload; ↔: no changes in physical performance; ↑: improved performance; ↓: impaired performance.

In a recent study, intraperitoneal administration of caffeine also provided evidence of the participation of DA in thermoregulatory adjustments induced by exercise and in modulating performance, as caffeine increased the concentration of this monoamine in the preoptic area (69). This increase in DA concentrations evoked by caffeine is probably a result of inhibition of central adenosine activity, which inhibits activity of the dopaminergic system. When treated with caffeine, rats exhibited prolonged time to fatigue and achieved higher core temperature values. Therefore, the authors suggested that the ergogenic effect of caffeine is due to an increase in central DA concentration, which prevents the development of fatigue (69). The main hypothesis is that the DA in the preoptic area blocked the signal for exercise cessation that resulted from the thermal overload, thereby increasing the tolerance to exertional heat strain (15,67). DA is likely involved in cutaneous heat dissipation, as blockade of CNS dopamine activity with SCH23390 (D_1_ receptor antagonist) and eticlopride (D_2_ receptor antagonist) prolonged hyperthermia after performing a fatiguing, incremental speed exercise, without prolonging metabolic activation (68).

The relationship between physical performance and DA has also been investigated in experiments with humans (Table 3). Administration of bupropion prolonged the time to fatigue during an exercise protocol in a cycle ergometer with constant power output, followed by an “against the clock” (time-trial) protocol, both performed in a hot environment (30°C). Improved performance was not observed during the same exercise in a temperate environment (18°C) (14). Because bupropion is a dual DA/NA reuptake inhibitor, it was imperative to understand the role of each neurotransmitter on fatigue. To achieve this purpose, the cyclists were given methylphenidate (20 mg), a specific inhibitor of DA reuptake (70). In this experiment, the subjects treated with methylphenidate showed improved performance and a greater increase in core temperature at 30°C, without changing their rating of perceived exertion (70). In fact, the higher tolerance to heat stress suggests an important effect of DA in motivation and fatigue, but it favors the occurrence of intestinal permeability and thus may represent a risk to health (4). It is important to note that increased performance in humans treated with an inhibitor of DA reuptake was observed in warm environments (30°C), but not in temperate environments (18°C) (70). This is an important difference between the findings in experiments with rats and human beings, since central injection of DA induced an increased time to fatigue in rats running at 22°C (15), which represents a temperate environmental for rats.

To clarify the role of NA on the ergogenic effects of bupropion, cyclists ingested reboxetine, a NA reuptake inhibitor, before being subjected to time trials at 18° and 30°C (19). Of note, NA is synthesized from the amino acid tyrosine (same pathway as for DA synthesis) and also influences motivation and motor behavior, thereby playing an important role on fatigue (10). Reboxetine reduced physical performance and modified hormone concentrations at both environments, thereby indicating a central effect of the drug (19). Collectively, these findings indicate that the noradrenergic system decreases performance and confirm results from previous studies that increased DA activity is important in improving performance.

**Figure 4.**
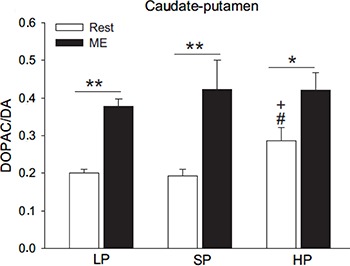
Concentrations of dopaminergic variables in the caudate–putamen at rest and after moderate-intensity exercise (ME) in rats with low (LP), standard (SP) and high (HP) performances. The figure shows the concentrations of 3,4-dihydroxyphenylacetic acid-to-dopamine (DOPAC/DA) ratio. Data are reported as means±SE. *P<0.05, **P<0.01 compared to rest.^+^P<0.05 compared to LP. ^#^P<0.05 compared to SP. This figure is reprinted with permission from Rabelo et al., 2015 (72).

The dopaminergic nigrostriatal pathway formed by the SNpc projections into the striatum is also associated with fine adjustment of movement (66,71). In this circuitry, DA modulates the direct and indirect pathways for the movement control, through interaction with D_1_ and D_2_ receptors, respectively (66). As increased DA in the CNS attenuated the decrease in mechanical efficiency during exercise (15), it is possible that the dopaminergic system adjusts motor responses that influence physical performance. Rats with high intrinsic predisposition to exercise exhibited higher basal dopaminergic activity in the caudate-putamen (dorsal striatum) (Figure 4), which could make them more efficient during treadmill running (72). This is a hypothesis to be tested in future studies. In addition to this motor response, because the activity of the striatal neurons is associated with positive reinforcement for exercise (71), it is also possible that increased striatal dopaminergic activity might induce greater motivation for physical exercise (72). Moreover, animals with brain lesions in this circuitry develop a hypoactivity frame due to the extinction of the positive reinforcement for exercise (73).

Chronic exercise induces plasticity in the dopaminergic pathways. These findings were observed in rodent models of Parkinson's disease (administration of 6-hydroxydopamine in rats and 1-methyl-4-phenyl-1,2,3,6-tetrahydropyridine in mice). In this condition of injury and degeneration of the nigrostriatal pathway, chronic exercise induced neural protection and recovery responses in the striatum, leading to improved motor control (74,75). Following chronic exercise, the motor deficit from the neuronal injury was reversed by restoration of DA concentrations and its metabolites in the striatum (75) and increased release of DA in the same area (74).

Such effects of chronic exercise on motor recovery and on the plasticity of the dopaminergic system have also been investigated in experiments with humans. Individuals with Parkinson's disease have increased grip strength and improved fine motor coordination after 12 weeks of training karate movements emphasizing the upper limbs (76), as well as improved walking patterns and body stability after treadmill training (77). It is possible that this increase in the motor performance after the exercise accompanied by attenuation of fatigue occurs as a result of an increased blood calcium concentration, which could lead to increases in dopaminergic activity (64).

From analysis and interpretation of all these studies, we conclude that the dopaminergic system influences physical performance by acting on different neural pathways, which include the control of movement, thermoregulation, perceived exertion, motivation and reward. Moreover, chronic exercise modulates the activity of this system, even in pathological conditions, such as Parkinson's disease.

## Interactions between the serotonergic and dopaminergic systems for determining fatigue

**Figure 5.**
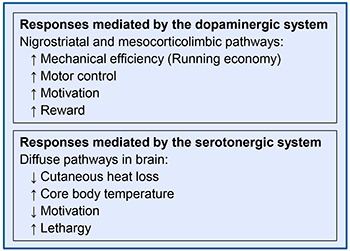
Motor and psycho-physiological effects induced by monoamines in the central nervous system that modulate fatigue during aerobic exercises.

As previously discussed, the development of fatigue is influenced by the neurotransmitters 5-HT and DA (Figure 5). In general, the activation of dopaminergic and serotonergic systems increased and decreased, respectively, the physical performance. However, these modulatory effects on fatigue may result from an interaction between these two neurotransmitters during exercise. There is evidence of 5-HT release inhibition by DA, as indicated by experiments showing increased and decreased serotonergic activity with the use of DA receptor antagonists and agonists, respectively (78). These findings were expanded later, when an inhibitory reciprocal relationship between the dopaminergic and serotonergic systems was demonstrated (27). These results allowed the reformulation of the “Central Fatigue Hypothesis”, which is now based on the relationship between 5-HT and DA. According to Davis and Bailey (79), fatigue is due to an increase in serotonergic activity and a decrease in dopaminergic activity. During physical exertion, a kinetic “pattern” develops in the activity of both systems, with gradual increases in serotonergic and dopaminergic activities being observed during the initial period of exercise. However, as exercise cessation approaches, the dopaminergic activity returns to basal values but the serotoninergic activity remains high (15,27,65,72).

The serotoninergic projections inhibit dopaminergic function in two different brain regions: the midbrain and the striatum/cortex. In the midbrain, stimulation of dorsal raphe serotonergic fibers causes the release of 5-HT in the SNpc (80), which is associated with a decrease in the firing rate of dopaminergic neurons, antagonizing the response mediated by DA (81). In this context, selective inhibitors of 5-HT uptake or agonists of 5-HT_1A_ receptors (at high doses) functionally inhibit nigral dopaminergic neurons (82,83), whereas anatomical and chemical injuries that destroy the raphe projections to the SNpc (82) or antagonists of 5-HT_2_ receptors (which tonically inhibit the mesencephalic dopaminergic system) (84) cause biochemical and functional disinhibition of the dopaminergic system.

In relation to the prosencephalon, immunohistochemical studies have shown that serotonergic neurons arising in the dorsal raphe nuclei are projected via the medial forebrain bundle to the striatum and cortex (85). Stimulation of these raphe striatal neurons or administration of 5-HT receptor agonists inhibits the neuronal firing rate in the striatum, presumably by decreasing release of DA in the synaptic cleft (86). Consistent with this inhibitory effect of 5-HT on DA release, lesions of the serotonergic projections induce disinhibition of the dopaminergic system and increase DA concentrations (87). Similar evidence exists for striatal control of limbic and cortical dopaminergic function (88).

In addition to the evidence demonstrating the inhibitory relationship between the two neurotransmitters and the influence of this relationship in physical exercise, there is also evidence that chronic exercise triggers plasticity in the neural pathways of 5-HT and DA (74). Physical training decreased the sensitivity of 5-HT receptors in rat substantia nigra (46), and prolonged the release of DA following the administration of methamphetamine in the caudate-putamen (89). Use of a running wheel for 6 weeks increased the expression of the mRNA for 5-HT_1A_, an autoreceptor that inhibits the synthesis and release of 5-HT, in the raphe (90); six weeks of wheel running also increased the mRNA for tyrosine hydroxylase in the SNpc and for D_2_ receptors in the caudate-putamen (66). When taken together, these modulations tend to enhance the activity of the dopaminergic system and to promote a concomitant down-regulation of the serotonergic system.

## Final remarks

Fatigue is a complex sensation involving changes in the CNS, which integrates information related to the motivation to exercise, external (environmental) conditions and internal conditions of the body, so as to prevent extreme physical exertion that may cause irreversible damage. This integration involves the action of neurotransmitters in the CNS. In general, studies with laboratory rodents and humans indicate that increased serotonergic activity and reduced dopaminergic activity are associated with accelerated fatigue. Further research should determine exactly how neurotransmitters, mainly 5-HT and DA, modulate fatigue during exercise, as well as elucidate whether physical training protocols induce plasticity of action of these neurotransmitters in order to improve physical performance.
